# Dynamic Contrast-Enhanced Ultrasound for Carotid Plaque Characterization: An Algorithm-Aware Technical Review

**DOI:** 10.3390/diagnostics16121808

**Published:** 2026-06-11

**Authors:** Nicola Morelli, Marco Spallazzi, Marina Biondi, Eugenia Rota, Lucia Mazza, Paolo Immovilli, Davide Colombi

**Affiliations:** 1 Neuroradiology Unit, Guglielmo da Saliceto Hospital, 29121 Piacenza, Italy; m.biondi@ausl.pc.it; 2 Neurology Unit, University of Parma, 43121 Parma, Italy; mspallazzi@gmail.com; 3 Neurology Unit, San Giacomo Hospital, 15067 Novi Ligure, Italy; eugenia.rota.md@gmail.com; 4 Neurology Unit, Guglielmo da Saliceto Hospital, 29121 Piacenza, Italy; l.mazza@ausl.pc.it (L.M.); p.immovilli@ausl.pc.it (P.I.); 5 Department of Diagnostic Imaging, Centro Diagnostico Rocca, 29121 Piacenza, Italy; colombidavide@gmail.com

**Keywords:** contrast-enhanced ultrasound, dynamic contrast-enhanced ultrasound, carotid plaque, intraplaque neovascularization, quantitative plaque characterization, imaging biomarkers, signal modeling

## Abstract

Carotid artery disease has traditionally been assessed according to luminal stenosis, although plaques with similar narrowing may differ substantially in biological activity and clinical risk. Intraplaque neovascularization is a key feature of plaque vulnerability, reflecting microvascular proliferation and its association with inflammation, hemorrhage, and structural destabilization. Dynamic contrast-enhanced ultrasound (DCE-US) offers a real-time, radiation-free method for evaluating intraplaque enhancement kinetics using strictly intravascular microbubble agents. However, its broader use in carotid plaque imaging remains limited by variability in acquisition protocols, contrast administration, signal processing, curve fitting, and parameter interpretation. This technical review clarifies the main analytical approaches used in carotid DCE-US, distinguishing bolus-based wash-in/wash-out analysis from destruction–replenishment modeling. Bolus analysis describes first-pass microbubble transit through the plaque microvasculature and commonly provides parameters such as peak intensity, wash-in slope, area under the curve, and time to peak. Destruction–replenishment analysis evaluates post-destruction refill under stable or quasi-stable contrast conditions and relies on model-based estimation of plateau intensity and the replenishment rate. Because these approaches interrogate different kinetic regimes, their outputs should not be considered interchangeable, even when similar terms are used across studies. Particular emphasis is placed on the operational meaning of quantitative and semi-quantitative parameters, the assumptions underlying curve modeling, and the methodological consequences of ROI placement, motion correction, acoustic settings, and fitting constraints. Rather than proposing a universal acquisition protocol, this article provides practical principles for acquisition, analysis, and reporting, helping radiologists, neuroradiologists, neurologists, and vascular imaging specialists understand the processing steps, algorithmic assumptions, and model-dependent choices underlying software-derived curves and parameters. By making this analytical layer more explicit, the review seeks to support a transparent, reproducible, and biologically coherent approach to quantitative carotid plaque characterization.

## 1. Introduction

For decades, carotid artery disease has been primarily assessed through the degree of luminal stenosis, a paradigm rooted in surgical trials and consolidated by guideline-driven risk stratification [1,2,3]. Although clinically effective in selected contexts, this approach does not fully explain the heterogeneous behavior of plaques with similar degrees of narrowing. Increasing evidence indicates that cerebrovascular risk is more closely related to plaque biology—including inflammation, intraplaque hemorrhage, *intraplaque neovascularization* (IPN), and microstructural instability—than to luminal geometry alone [4,5].

This evolving understanding has shifted carotid imaging beyond purely morphological descriptors toward techniques capable of probing biological activity. Against this background, contrast-enhanced ultrasound (CEUS) has emerged as a particularly suitable modality [6]. Microbubble contrast agents remain strictly intravascular and enable real-time visualization of microvascular networks, offering direct insight into processes associated with plaque inflammation and destabilization. In particular, IPN—reflecting proliferation of microvessels arising predominantly from the adventitial vasa vasorum—has been consistently associated with plaque vulnerability and stroke risk in histopathological and imaging studies and is increasingly incorporated into imaging-based risk stratification approaches [7,8].

Contemporary approaches are therefore moving toward structured, biology-informed reporting systems, including Carotid Plaque-RADS, which aim to integrate compositional and vulnerability features beyond stenosis severity alone [9]. In this context, quantitative vascular imaging biomarkers may support more standardized plaque characterization, provided that acquisition strategies, analytical assumptions, and parameter definitions are clearly understood. Although CEUS is intrinsically dynamic, its clinical application in carotid imaging has traditionally relied on qualitative visual assessment of enhancement patterns rather than on explicit analysis of signal behavior over time.

When acquisition protocols are specifically designed for time-resolved signal sampling, *dynamic contrast-enhanced ultrasound* (DCE-US) allows for characterization of intraplaque contrast kinetics. This provides an in vivo surrogate of IPN-related enhancement and adds functional information to conventional plaque imaging.

Despite its pathophysiological rationale and favorable safety profile, quantitative DCE-US has not achieved widespread clinical adoption in carotid imaging. This limitation is not primarily due to technical feasibility or biological relevance but to persistent conceptual and methodological ambiguity. Variability in acquisition strategies, analytical models, and parameter interpretation has hindered standardization, particularly when metrics derived from different analytical paradigms are treated as interchangeable.

The aim of this technical review is therefore not to provide a comprehensive or systematic review of clinical outcome studies but to clarify how quantitative carotid ultrasound parameters obtained after contrast administration should be interpreted. Because acquisition and analysis remain strongly influenced by the ultrasound platform, this article is not intended as a universal protocol. Instead, it focuses on what lies behind dynamic enhancement curves and software-derived parameters, including signal processing, region-of-interest selection, curve fitting, and assumptions related to the mathematical model. By distinguishing bolus *wash-in/wash-out* analysis from *destruction–replenishment* modeling, it aims to help clinicians understand what these curves and parameters represent, what they do not represent, and why they require separate interpretation.

## 2. Ultrasound Contrast Agent and Administration

Dynamic contrast-enhanced ultrasound employs intravascular microbubble contrast agents. In routine clinical practice, a widely used *third-generation agent* consists of *sulfur hexafluoride gas* stabilized by a phospholipid shell [10]. It is supplied as a lyophilized powder and reconstituted immediately before use with saline to obtain a homogeneous microbubble suspension.

For carotid plaque imaging, contrast administration is commonly performed as an intravenous bolus of 2.4 mL of sulfur hexafluoride microbubble contrast through a peripheral vein, followed by a saline flush. Practical recommendations describe bolus injection at approximately 1–2 mL/s, avoiding excessive pressure to reduce the risk of microbubble disruption, followed immediately by a 5–10 mL saline flush. A three-way stopcock may be used to allow for sequential administration of contrast and saline without disconnecting the syringes, reducing handling delay and preserving a compact bolus profile.

Compared with earlier formulations, third-generation microbubble agents provide greater stability and a more consistent acoustic response during low-mechanical-index imaging while remaining confined to the vascular compartment [10]. Earlier agents showed shorter persistence and less reproducible contrast signals, limiting their suitability for quantitative applications.

For dynamic studies, the contrast agent should preferably be administered shortly after preparation and injected without delay after withdrawal. If administration is delayed, brief re-agitation of the vial before withdrawal helps restore suspension homogeneity. From a *safety perspective*, sulfur hexafluoride microbubbles are eliminated independently of renal function, through pulmonary exhalation of the gas component and endogenous metabolism of the phospholipid shell; accordingly, no dose adjustment is required in patients with renal impairment [10]. Because dynamic quantification is sensitive to contrast delivery, microbubble persistence, and temporal sampling, preparation and injection should be reported consistently. These factors influence the shape of dynamic enhancement curves and, consequently, the interpretation of quantitative parameters.

## 3. Physical Principles and Signal Processing

Contrast-enhanced ultrasound exploits the *nonlinear acoustic behavior* of intravascular microbubbles during low-mechanical-index insonation. Unlike tissues, which mainly generate linear backscatter at the transmitted fundamental frequency, microbubbles oscillate asymmetrically in response to acoustic pressure and generate *harmonic* and *subharmonic* components that can be selectively detected for contrast-specific imaging [11].

To isolate these nonlinear signals, ultrasound systems use dedicated transmission and reception schemes with real-time processing, including pulse inversion, amplitude modulation, and hybrid techniques [12]. Vendor-specific implementations increase microbubble sensitivity while suppressing tissue signal. Imaging is usually performed at a low mechanical index to preserve microbubble integrity and allow for repeated sampling.

Two main strategies can be used to characterize contrast behavior: bolus *wash-in/wash-out* analysis (WI/WO) and *destruction–replenishment* modeling (DR). In WI/WO, continuous low-mechanical-index imaging follows an injected microbubble bolus. In DR, a brief high-mechanical-index pulse destroys microbubbles within the imaging plane, and subsequent low-mechanical-index imaging records signal recovery as circulating microbubbles refill the region of interest.

Quantitative DCE-US requires contrast-specific low-mechanical-index *harmonic imaging*, dynamic cine-loop storage, and appropriate quantification software. For DR, the system should also provide a high-mechanical-index *flash* or destruction function. Dynamic analysis is based on *time–intensity curves* (TICs), which graphically reconstruct contrast signal changes over time within a selected region of interest (ROI). TICs may be generated on the ultrasound platform or from exported DICOM cine-loop data using external software.

Because these outputs are shaped by acquisition settings, signal processing, and software implementation, they should be interpreted as semi-quantitative descriptors rather than absolute perfusion measurements. This limitation is particularly relevant in plaque imaging, where small sampling regions, weak enhancement, acoustic shadowing, and motion may amplify variability across systems and analysis pipelines.

A key technical issue concerns signal transformation before image display and analysis. After insonation, backscattered echoes are converted into radiofrequency signals spanning a wide dynamic range. *Logarithmic compression* maps this range into visually displayable intensities [13]. However, TIC analysis requires data in a *linear signal domain*, because display compression alters the relationship between signal intensity and microbubble concentration. *Signal linearization* is therefore necessary for quantitative interpretation; without it, brightness changes may not correspond proportionally to changes in microbubble concentration.

Acquisition parameters, including mechanical index, frame rate, flash characteristics, recording duration, motion correction, and ROI strategy, influence curve shape and output stability [14,15,16,17,18,19]. Although both approaches rely on TIC generation, WI/WO and DR differ in acquisition design, kinetic assumptions, and mathematical fitting. WI/WO describes *first-pass transit* of an injected microbubble bolus, whereas DR evaluates *post-destruction signal recovery* under stable or quasi-stable intravascular conditions. Their outputs should therefore be interpreted within their respective kinetic contexts, rather than as equivalent measures of the same plaque property.

**Figure 1:**
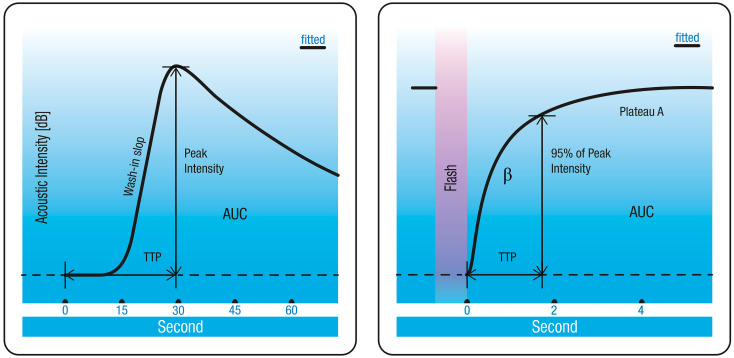
Schematic comparison between bolus-based wash-in/wash-out analysis and destruction–replenishment modeling. (**Left panel**): in WI/WO analysis, continuous low-mechanical-index imaging follows the first-pass transit of a compact microbubble bolus through the plaque microvasculature. The resulting curve provides descriptors such as wash-in slope, peak intensity, time to peak, and area under the curve. (**Right panel**): in DR analysis, after stable or quasi-stable contrast conditions are reached, a brief high-mechanical-index pulse destroys microbubbles within the imaging plane, and subsequent low-mechanical-index imaging records post-destruction refill. Replenishment is commonly modeled with a mono-exponential function, in which plateau intensity (**A**) represents the asymptotic refill signal and β describes the replenishment rate. In this setting, time to peak is model-derived rather than directly observed. The vertical shaded band indicates the high-mechanical-index destruction pulse, temporally expanded for illustrative purposes. The short horizontal segment marks this pre-destruction steady-state signal, whereas the dashed horizontal line represents the baseline reference.

An overview of the operational meaning of commonly reported parameters is provided in Table 1. The relationship between the two approaches and their respective TIC behavior is illustrated in Figure 1.

## 4. Contrast-Enhanced Ultrasound Acquisition and Analysis

### 4.1. Bolus-Based Wash-In/Wash-Out Analysis

In bolus DCE-US, contrast kinetics are driven by rapid intravenous injection of a compact microbubble bolus, followed by continuous *low-mechanical-index* imaging during its *first passage* through the vascular system. In carotid applications, acquisition is usually continued for approximately 60–90 s to capture both wash-in and wash-out phases. Cine-loop recording is performed using a dedicated contrast mode, with frame rates sufficient to sample early enhancement, commonly around 10–20 frames per second depending on imaging depth and system settings.

Imaging is performed on a single plaque plane, either axial or longitudinal, selected according to plaque visibility and spatial stability. Signal intensity is then extracted offline by placing a predefined *region of interest* (ROI) on the stored sequence, and the mean ROI signal over time is used to generate a TIC that graphically represents contrast wash-in and wash-out within the plaque microvasculature. Dual display with contrast imaging and grayscale B-mode may help plaque localization and improve ROI consistency during post-processing.

Bolus analysis is sensitive to injection profile, systemic circulation, and acquisition timing; therefore, complete capture of the first-pass event is essential. Short or incomplete recordings may still allow for curve fitting, but the resulting values may not reflect the underlying contrast kinetics in a biologically meaningful way. Reports should specify the synchronization between contrast injection and recording, total acquisition duration, frame rate when available, and criteria for defining a technically adequate curve.

In 2D acquisitions, *frame-to-frame in-plane motion correction* is usually performed using image-registration or tracking algorithms, whereas out-of-plane motion remains a major limitation. ROI selection also influences curve shape: circular, elliptical, or polygonal contours may sample different plaque regions, while areas close to the lumen should be avoided to reduce partial volume effects and acoustic blooming.

Several mathematical functions have been proposed to describe bolus TICs, differing in their assumptions about contrast dispersion, transit time distribution, and robustness to noisy or incomplete data. With commercial software, implementation details such as baseline handling, smoothing, parameter limits, and convergence rules may differ across platforms and should be considered when interpreting quantitative outputs. The main *bolus fitting models* are summarized in Table 2 and discussed below, with emphasis on their practical interpretation in carotid plaque imaging.

#### 4.1.1. Log-Normal Model

The log-normal model is so named because transit times approximate a *normal* distribution after *logarithmic* transformation. In the original time domain, the bolus TIC remains asymmetric, with a rapid rise followed by slower wash-out, reflecting dispersed microbubble arrival and clearance within the plaque ROI. Thus, logarithmic transformation facilitates mathematical fitting without implying that the displayed curve is visually symmetric.

Both *wash-in-only* and *full-curve* implementations are used. The former restricts fitting to the rising phase, whereas the latter describes signal evolution across both arrival and clearance. The practical value of the log-normal model lies in its numerical stability, tolerance to noise, and adaptability to clinical data.

Common outputs include peak intensity, wash-in slope, area under the curve, and time to peak. Amplitude-related values describe signal magnitude and the extent of contrast transit, whereas temporal descriptors are influenced by local microvascular features and extrinsic factors, including injection profile and systemic hemodynamics. Thus, log-normal outputs are best viewed as first-pass transit metrics rather than standalone perfusion measures.

#### 4.1.2. Gamma-Variate Models

Gamma-variate fitting uses a mathematical function designed to represent the time course of bolus passage as an asymmetric curve, with signal rise, peak enhancement, and subsequent wash-out. Widely used within indicator dilution modeling, this approach is frequently applied to fit asymmetric TICs during bolus passage and may provide stable fitting when the first-pass event is well captured.

However, its physiological interpretation requires caution. The model implicitly assumes relatively ordered transport, simplified vascular geometry, and a coherent relationship between contrast arrival and clearance. Such assumptions may be difficult to reconcile with carotid plaque neovascularization, where microvessels are typically tortuous, irregular, spatially heterogeneous, and variably connected to the adventitial vasa vasorum. As a result, the fitted curve may describe the observed signal adequately, but its physiological specificity as a direct measure of plaque perfusion should be considered limited in routine carotid applications.

#### 4.1.3. Diffusion-with-Drift/Local Density Random Walk Models

Diffusion-with-drift models, including local density random walk formulations, describe microbubble transport as the combined effect of *directional flow* and *random dispersion*. The directional component reflects organized movement along vascular pathways, whereas dispersion accounts for bolus spreading due to heterogeneous transit times and variable flow paths. This concept is appealing for carotid plaque imaging, because plaque neovascularization behaves as a disorganized microvascular network rather than a uniform vascular compartment.

Their main practical limitation is the need for data quality sufficient to separate flow-related from dispersion-related components. When this condition is not met, different parameter combinations may fit the same TIC, reducing identifiability and making estimates unstable. For this reason, diffusion-with-drift and local density random walk models remain conceptually informative, but their routine value in carotid plaque imaging is currently limited.

#### 4.1.4. Lagged and Compartmental Models

Lagged-normal models introduce an explicit *lag*, defined as the delay between contrast injection or systemic arrival and measurable enhancement within the plaque ROI. By separating this arrival delay from the subsequent transit phase, these models attempt to distinguish delayed contrast access from the shape of the enhancement curve itself.

This distinction may be useful in carotid plaques with delayed intraplaque enhancement. However, estimating a separate lag parameter increases model complexity and requires a clearly captured early enhancement phase; otherwise, the lag estimate may become unstable and affect the remaining fitted parameters.

*Multi-compartment formulations* assume that the observed signal arises from vascular components with distinct transit properties. Although this may better reflect plaque heterogeneity, reliable fitting requires data quality rarely available in routine carotid imaging, so their use remains mainly experimental.

Taken together, bolus analysis provides a descriptive assessment of first-pass contrast transit through the plaque microvasculature. Its strength lies in capturing global enhancement dynamics, whereas its limitations reflect sensitivity to injection conditions, systemic circulation, recording duration, and curve completeness. Interpretation should therefore remain linked to the fitting function and acquisition conditions used.

**Figure 2:**
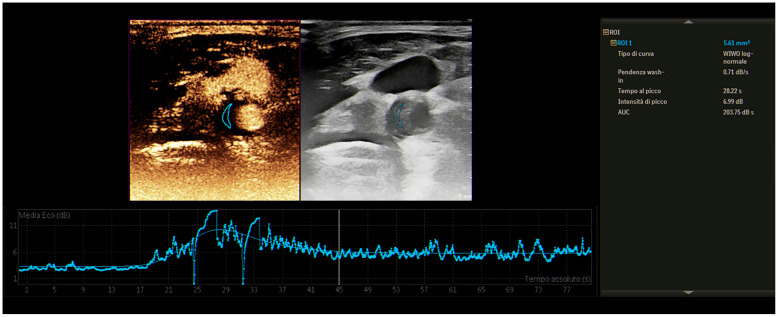
*Case-based extraction of dynamic signal behavior using bolus-based wash-in/wash-out analysis.* Representative WI/WO TIC analysis of an internal carotid artery plaque. Axial dual-display images show contrast-specific imaging alongside the corresponding grayscale B-mode image. The curvilinear blue ROI is positioned within the deeper portion of the plaque, avoiding luminal contamination. TIC analysis, performed after automated in-plane motion correction, depicts bolus passage fitted using a log-normal model, yielding semi-quantitative parameters including wash-in slope, time to peak, peak intensity, and area under the curve. Although the curve remains visually asymmetric, fitting is performed in the logarithmic time domain. The thicker oscillating blue trace represents the raw frame-by-frame contrast-enhanced ultrasound signal dominated by microbubble backscatter, whereas the smooth blue curve corresponds to the fitted model. The vertical white marker indicates the cine-loop time point corresponding to the displayed B-mode and contrast-specific ultrasound frame.

A representative case, including ROI placement, motion correction, log-normal fitting, and parameter extraction, is shown in Figure 2.

### 4.2. Destruction–Replenishment Analysis

DR analysis was developed to reduce the influence of injection profile and systemic circulation, two intrinsic limitations of bolus-based methods. Rather than describing transient first-pass contrast transit, it evaluates local microvascular refill after deliberate microbubble destruction.

In its ideal implementation, *true steady-state* DR is performed under stable intravascular contrast conditions, so that signal recovery mainly reflects local refill kinetics. EFSUMB technical recommendations describe continuous infusion of sulfur hexafluoride microbubble contrast at 1 mL/min or less, with up to two vials, corresponding to 9.6 mL, in selected studies [14,15]. This volume provides about 10 min of administration at 1 mL/min and about 20 min at 0.5 mL/min. After stabilization, microbubbles are deliberately destroyed within the imaging plane, and subsequent recovery is recorded as the ROI is refilled by circulating contrast. However, the need for homogeneous slow infusion and dedicated equipment, such as a *rotating pump* to maintain contrast suspension, limits the practicality of true steady-state in routine examinations. In pragmatic protocols, DR is often performed after a short delay, commonly within 2–5 min after bolus injection, when the residual contrast signal appears sufficiently stable, representing a bolus-derived *quasi-steady-state* compromise.

Technically, DR relies on low-mechanical-index contrast imaging before and after a brief high-mechanical-index flash, which disrupts microbubbles within the insonated plane. In carotid applications, very low MI imaging in the range of 0.05–0.10 is commonly used, followed by high MI destruction pulses, often around 0.7–1.0, with a flash duration of about 200 ms. Post-flash recovery is usually recorded for 15–25 s to capture early refill. These values should be regarded as representative ranges rather than fixed requirements, because optimal settings depend on the ultrasound platform and acquisition conditions.

DR also differs from bolus analysis in cine-loop handling. Bolus analysis evaluates the full passage of contrast through the plaque, whereas DR requires identification of the destruction event and analysis of the recovery segment only. Including pre-flash frames violates the assumptions of the refill model and may distort parameter estimation; therefore, the analyzed segment should begin immediately after the flash frame.

#### 4.2.1. Mono-Exponential Replenishment Model/Wei Model

The mono-exponential model originally described by Wei and colleagues [20] represents the most widely used and operationally robust formulation for destruction–replenishment analysis. It describes post-destruction signal recovery as a *first-order* exponential increase toward a plateau:

I(t) = A · (1 − e^−βt^)

where I(t) is the contrast signal intensity at time t; A is the fitted plateau, often used operationally as “peak intensity”; and β is the replenishment rate constant, frequently labeled as “wash-in slope”. The curve rises rapidly after microbubble destruction and progressively slows as it approaches A, reflecting gradual refilling of the insonated microvascular space. Plateau intensity A is interpreted as a surrogate of relative *microvascular blood volume*, whereas β describes refill rate and is associated with *flow-related* properties. The mono-exponential model is simple, numerically stable, and compatible with short acquisitions, usually 10–25 s, allowing for repeated measurements without additional contrast administration. However, it remains a simplified representation of the irregular and heterogeneous neovascular network of vulnerable carotid plaques.

Terminology is a common source of misinterpretation. In DR analysis, “peak intensity” corresponds to the fitted plateau A rather than to a directly observed transient maximum. Similarly, “time to peak” is usually defined as the time required to reach a predefined fraction of the plateau, most often 95% of A. The output labeled as “wash-in slope” should not be interpreted as the bolus-derived wash-in slope; in this context, it reflects the refill rate constant or the early slope of the fitted recovery curve.

In summary, mono-exponential DR parameters describe post-flash refill behavior. Additional formulations addressing vascular heterogeneity, acoustic field effects, and spatially variable replenishment are outlined in the following sections. These methods may improve physiological plausibility but usually require higher-quality data and processing tools not routinely available in carotid plaque imaging.

#### 4.2.2. Advanced Replenishment Models

*Multivessel models.* Multivessel formulations extend the mono-exponential approach by allowing signal recovery to arise from vascular components with different refill rates rather than from a single homogeneous compartment. This may better reflect plaque heterogeneity, where regions with faster and slower filling can coexist within the same lesion.

*Acoustic field-corrected models*. Other formulations account for differences between the low-mechanical-index imaging field and the high-mechanical-index destruction field. Because beam profile, local pressure, and attenuation can influence both microbubble disruption and the apparent recovery curve, these corrections may reduce technical bias but require detailed knowledge of system behavior.

*Geometry-informed perfusion models*. More advanced models incorporate assumptions about microvascular architecture and spatial contrast distribution to approximate true microvascular perfusion more closely. However, these approaches overlap conceptually with multivessel and acoustic field-corrected models, and their need for calibration, controlled acquisition, and advanced processing limits their current use mainly to research settings.

**Figure 3:**
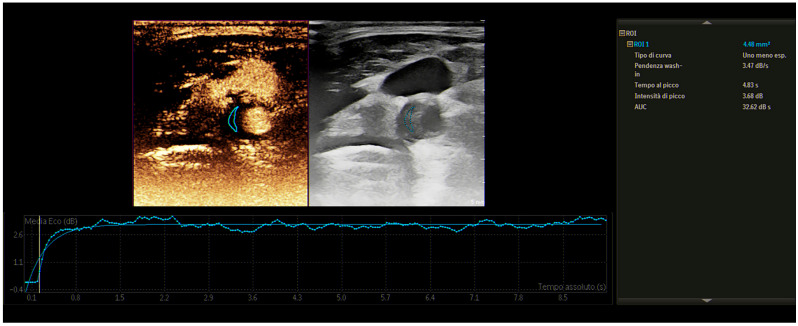
*Case-based extraction of dynamic signal behavior using destruction–replenishment analysis*. Representative DR TIC analysis of an internal carotid artery plaque. Contrast-specific images acquired after operator-activated high-mechanical-index microbubble destruction show the post-flash replenishment phase. After automated motion correction, the continuous blue curve represents the mono-exponential fitted model, yielding semi-quantitative parameters including replenishment slope, time to peak, plateau intensity, and area under the curve. Analysis starts immediately after the flash; therefore, the initial signal increase reflects post-destruction microbubble refill rather than first-pass bolus transit. The curvilinear blue ROI marks the plaque region selected for TIC analysis. The oscillating blue trace represents the raw frame-by-frame contrast-enhanced ultrasound signal, dominated by microbubble backscatter and background signal fluctuations.

The principal destruction–replenishment models, together with their assumptions and practical trade-offs in carotid plaque imaging, are summarized in Table 3. A representative example of quasi-steady-state destruction–replenishment analysis is shown in Figure 3.

## 5. Discussion

### 5.1. From Signal Modeling to Plaque Biomarkers

Moving from stenosis-based assessment toward biologically informed plaque characterization has increased interest in DCE-US for evaluating intraplaque microvascular behavior [4,21,22]. In this context, its clinical value depends on understanding what each acquisition and analysis strategy actually measures.

Published evidence remains heterogeneous, with studies using visual assessment, binary or ordinal scores, descriptions of peripheral, central, or diffuse enhancement, and TIC-based analysis [21,22,23,24,25,26,27,28,29,30,31]. This variability limits cross-study comparison and the definition of reproducible thresholds for clinical or research use.

For biomarker interpretation, the distinction between bolus WI/WO and DR remains clinically relevant. Bolus analysis captures first-pass contrast transit, whereas DR focuses on post-destruction refill under stable or quasi-stable contrast conditions. These approaches emphasize different aspects of microvascular access, organization, and filling behavior. Clinically, this matters because IPN may vary across contrast conditions. Some plaques may show *early* first-pass enhancement, whereas others may be better characterized by *slower* post-destruction refill. Divergent WI/WO and DR results should therefore be interpreted as potentially reflecting different microvascular behaviors, rather than automatically regarded as technical inconsistency.

Table 4 summarizes a practical example of sequential integration of WI/WO and DR within the same examination.

WI/WO and DR can be used *separately* or *sequentially* within the same examination, depending on equipment, contrast administration, and patient cooperation [32]. WI/WO requires continuous recording of bolus transit and is more vulnerable to timing errors, motion, and incomplete cine-loop acquisition. DR provides a shorter, more locally weighted assessment of post-destruction refill and may be useful when enhancement is weak, delayed, or heterogeneous. These differences also have practical implications for plaque selection and repeated acquisitions in patients with multiple lesions.

The aim is not to obtain two estimates of the same quantity but to sample different kinetic behaviors. Direct numerical comparison between WI/WO and DR parameters should therefore be avoided, because differences in peak or plateau intensity, AUC, or time to peak may reflect acquisition design, contrast kinetics, recording duration, and intravascular microbubble concentration rather than true differences in plaque vascularity. For this reason, acquisition conditions, fitting models, and operational definitions should remain distinct.

From a *clinical perspective*, contrast-enhanced quantitative assessment may be most useful in carotid plaques with intermediate stenosis, approximately 50–69%, where lesion size usually allows for more reliable intraplaque sampling and stenosis severity alone may be insufficient to guide management. In lower-grade stenoses, partial volume effects and adjacent luminal contrast may limit quantification. When technically feasible, IPN assessment may still add biological and prognostic information within structured systems such as Carotid Plaque-RADS [33].

### 5.2. Practical ROI Placement and Plaque Selection in Challenging Scenarios

Several practical issues should be considered when applying quantitative contrast-enhanced analysis to routine carotid plaque imaging. No universally accepted ROI size or fixed plaque-volume proportion has been established. ROI placement should therefore be adapted to plaque size, morphology, acoustic accessibility, and the analytical objective [19]. The scanning plane should provide the best available acoustic window and spatial stability while sampling the most informative analyzable plaque portion. Because 2D ultrasound interrogates only one plane, some plaque regions are inevitably excluded from analysis.

For *intraplaque quantification,* the ROI should remain within analyzable plaque tissue throughout the cine-loop, avoiding both the luminal interface and the outer plaque boundary. Carotid pulsatility, respiration, and probe-related motion may shift the plaque relative to the ROI. Although in-plane motion correction can reduce frame-to-frame displacement, it cannot fully prevent contamination from luminal contrast, acoustic blooming, or out-of-plane motion. As a practical empirical approach, small intraplaque ROIs are often preferable and, when plaque size allows, should not exceed approximately one third of plaque thickness; however, this should be regarded as a pragmatic rule rather than a guideline-based threshold. Multiple ROIs may be sampled retrospectively on the same cine-loop, for example in proximal, middle, and distal plaque regions, to explore spatial heterogeneity. Alternatively, ROI placement may be targeted to the region showing the greatest focal intraplaque contrast enhancement, although this strategy may miss enhancement that is not visually conspicuous.

*Calcification* is a major limitation for ROI-based analysis. Heavily calcified plaques, including Gray-Weale–Geroulakos type V plaques, may produce acoustic shadowing that prevents reliable assessment of IPN [21]. Superficial, circumferential, or shell-like calcification is particularly problematic because contrast-enhanced imaging cannot recover information from shadowed plaque components. If calcification is focal, alternative insonation planes, including non-standard probe angulations, should be attempted to identify a visible plaque segment suitable for analysis. When the plaque interior remains obscured, quantification should be limited to the visible portion, or the plaque should be considered unsuitable for reliable analysis. This limitation should be stated in the report.

In patients with *multiple plaques*, plaque selection depends on the acquisition strategy. For bolus-based WI/WO, the first-pass bolus can be optimally captured only once for a selected scanning plane. Therefore, the initial acquisition should target the plaque with the most suspicious morphology, such as heterogeneous echotexture with echolucent components, surface irregularity, or ulceration, provided that it remains technically accessible for reliable analysis. Subsequent WI/WO acquisitions on additional plaques may be confounded by residual intravascular contrast and loss of a compact bolus profile. Conversely, DR protocols performed after stable or quasi-stable intravascular contrast conditions have been reached may allow for repeated assessment without additional contrast administration [14,15,32]. Multiple cine-loops can then be acquired after separate high-MI destruction pulses, either on different portions of the same plaque or on different plaques, within the available post-bolus contrast window and provided that circulating contrast remains sufficiently stable for repeated refill assessment. This makes DR more flexible than WI/WO for evaluating plaque heterogeneity or multiple lesions, although at the cost of longer examination and post-processing time.

### 5.3. Curve Interpretability, Multiparametric Integration, and Future Directions

Absence of a clear TIC should not be automatically equated with absence of intraplaque microvascularization. It may reflect truly undetectable enhancement but also motion artifacts, inadequate acoustic window, weak contrast signal, suboptimal timing, incomplete recording, or local factors preventing stable probe positioning, such as severe calcification, short neck anatomy, or local devices. In these cases, descriptive interpretation or exclusion from quantitative analysis is preferable to forced parameter extraction.

**Figure 4:**
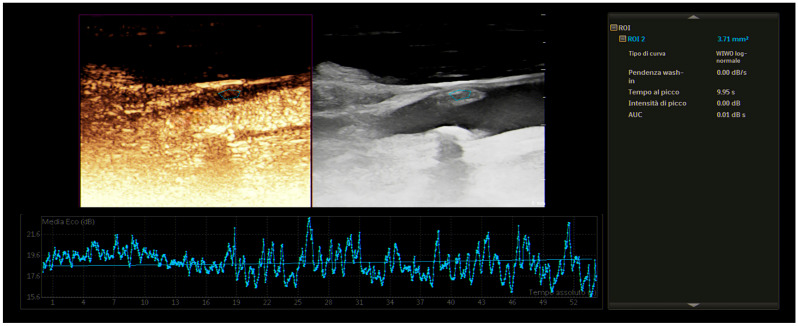
*Negative perfusion result on bolus-based wash-in/wash-out log-normal analysis*. Negative perfusion result on bolus-based wash-in/wash-out log-normal analysis. TIC analysis of an internal carotid plaque with curvilinear blue ROI placement on the grayscale B-mode image. The oscillating blue trace represents the raw frame-by-frame contrast-enhanced ultrasound signal, which remains close to background noise. The continuous blue line represents the fitted log-normal model and shows no meaningful contrast-related increase, supporting absence of measurable intraplaque enhancement. This pattern should be distinguished from technically inadequate acquisitions.

A representative example of WI/WO log-normal analysis with absent measurable intraplaque enhancement is shown in Figure 4.

When a technically interpretable TIC is present, extracted values remain meaningful only in relation to the acquisition strategy and fitting method used. In WI/WO analysis, this generally corresponds to a recognizable bolus-related enhancement curve above background noise, whereas in DR analysis it corresponds to a post-destruction refill curve compatible with mono-exponential recovery. A separate luminal ROI may serve as a technical reference for bolus arrival and intravascular contrast enhancement, while plaque quantification should remain based on the intraplaque ROI.

Dynamic contrast assessment should be viewed as one component of multiparametric plaque evaluation. CT and MRI *vessel wall imaging* characterize structural and compositional features, such as morphology, calcification, lipid-rich necrotic core, and intraplaque hemorrhage [29], whereas *elastography* assesses mechanical properties and tissue heterogeneity. In this broader context, dynamic ultrasound captures intraplaque microvascular behavior, complementing rather than duplicating morphologic imaging.

Future studies should prioritize methodological consistency before broad clinical translation. Multicenter validation should address acquisition reproducibility, inter-platform variability, curve quality, and the biological or prognostic meaning of quantitative outputs. Only then can these metrics evolve from descriptive parameters into reliable vascular imaging biomarkers.

*Artificial intelligence* may further support quantitative carotid plaque assessment by integrating radiomic and texture-based analysis of conventional ultrasound features [34,35,36,37]. In contrast-enhanced ultrasound, *machine learning* and *deep learning* methods have been applied to plaque vulnerability classification, automated segmentation, and neovascularization grading [38,39]. These developments build on earlier quantitative approaches to IPN and spatio-temporal enhancement analysis [24,40,41]. Overall, current evidence remains mainly focused on plaque characterization and vulnerability classification, whereas direct AI-based modeling of dynamic perfusion curves is still less developed [42]. In this setting, these methods could help standardize region selection, reduce operator dependence, and extract high-dimensional enhancement features that complement structural plaque descriptors. However, external validation and careful assessment of feature stability are still needed before such outputs can be translated into reliable imaging biomarkers.

## 6. Conclusions

Dynamic contrast-enhanced ultrasound offers a functional perspective on carotid plaque biology by assessing intraplaque microvascular behavior beyond luminal stenosis. Its quantitative value depends on understanding how WI/WO and DR reflect different kinetic behaviors, and how acquisition, ROI placement, plaque selection, motion correction, and model-dependent choices shape the resulting parameters. A disciplined, context-aware interpretation of these metrics, together with explicit reporting of technical limitations such as acoustic shadowing and incomplete plaque sampling, may strengthen carotid plaque characterization and support future validation of contrast-based vascular biomarkers.

## Figures and Tables

**Table 1:** Software-reported TIC-derived parameters and operational interpretation.

Paradigm	Parameter	Derivation Method	Operational Meaning	Unit Reported
*Wash-in/wash-out approach*	Peak intensity	Maximum signal recorded during bolus passage	Signal amplitude during *first-pass* contrast transit	dB *
	Wash-in slope	Initial rate of signal increase at bolus arrival	Speed of contrast inflow	dB/s *
	Area under the curve (AUC)	Time-integrated signal over the wash-in/wash-out phase	Overall extent and duration of contrast transit	dB·s *
	Time to peak (TTP)	Time interval from baseline to maximal signal	Temporal descriptor of bolus passage	s
*Destruction–replenishment approach*	Peak intensity	Plateau level estimated by exponential fitting	Asymptotic refill signal (parameter A)	dB *
	Wash-in slope	Initial slope of post-destruction signal recovery	Replenishment rate constant (parameter β)	dB/s *
	Area under the curve (AUC)	Integral of the fitted refill curve	Global refill behavior over time	dB·s *
	Time to peak (TTP ^†^)	Time required to reach 95% of the fitted plateau	Model-derived descriptor of refill kinetics	s

* Signal-intensity values are expressed in system-dependent units resulting from logarithmic compression and internal scaling. ^†^ In destruction–replenishment analysis, TTP corresponds to the time needed to reach 95% of the estimated plateau and does not represent a true signal maximum.

**Table 2:** Bolus-based DCE-US analytical models (WI/WO).

Model	Underlying Assumptions	Main Strengths and Limitations
*Log-normal*	*First-pass* microbubble transit times follow an asymmetric distribution that approximates a Gaussian shape in logarithmic time	Robust to noise and incomplete curves; suitable for heterogeneous plaque microvasculature. Semi-quantitative and influenced by bolus injection and systemic circulation.
*Gamma-variate*	*Indicator-dilution behavior* with relatively ordered transport pathways	Efficient curve fitting; limited physiological plausibility in complex plaque neovascularization and sensitive to acquisition quality.
*Lagged-normal*	Contrast arrival *delay* can be separated from subsequent transit kinetics	Explicitly accounts for delayed bolus arrival; susceptible to instability and overfitting in routine carotid imaging.
*Diffusion-with-drift (LDRW—Local Density Random Walk)*	Contrast transport results from the combination of *directed flow* and *random dispersion*	Conceptually appealing; limited parameter identifiability with typical clinical temporal resolution and signal-to-noise ratio.
*Multi-compartment bolus models*	The observed signal arises from *multiple vascular compartments* with distinct transit characteristics	Higher theoretical detail; rarely supported by routine plaque imaging data due to limited temporal resolution and signal quality.

**Table 3:** Destruction–replenishment analytical models and advanced extensions.

Model	Underlying Assumptions	Main Strengths and Limitations
Mono-exponential (Wei)	Post-destruction signal recovery follows first-order kinetics toward a single asymptotic level	Stable, practical, and compatible with short acquisitions; less dependent on bolus timing. Simplifies the spatial and kinetic heterogeneity of plaque neovascularization.
Multivessel/geometric	Refill may reflect vascular components with different kinetics and spatial distributions.	Greater physiological plausibility but requires higher data quality, calibration, and complex fitting; mainly suited to research settings.
Acoustic field-corrected	Differences between low- and high-MI fields may affect microbubble disruption and apparent recovery.	Reduces bias related to mechanical index and field non-uniformity; system-specific and highly sensitive to acquisition settings.

**Table 4:** Illustrative sequential contrast-enhanced ultrasound workflow.

Phase	Operational Workflow	Recording Window	Primary Analytical Model	Software-Reported Outputs
*Wash-in/wash-out*	Intravenous bolus of 2.4 mL microbubble contrast followed by saline flush; continuous low-MI imaging on a fixed plaque plane; plaque ROI defined after acquisition	~60–90 s	Log-normal curve fitting	Peak intensity, Wash-in slope, AUC, TTP
*Destruction–replenishment*	After ~5 min, when residual contrast signal appears *quasi-stable*, high-MI destruction pulse is applied; low-MI imaging records the post-flash refill segment	~10–25 s	Wei mono-exponential refill model	Peak intensity (plateau A), Wash-in slope (rate β), AUC (fitted curve), TTP (time to 95% of plateau, if reported)

## Data Availability

No new datasets were generated or analyzed for the purposes of this technical review.

## Abbreviations

The following abbreviations are used in this manuscript:

AUC	Area under the curve
CEUS	Contrast-enhanced ultrasound
DCE-US	Dynamic contrast-enhanced ultrasound
DR	Destruction–replenishment
IPN	Intraplaque neovascularization
MI	Mechanical index
ROI	Region of interest
TIC	Time–intensity curve
TTP	Time to peak
WI/WO	Wash-in/wash-out
